# The improved dynamic slicing for spectrum-based fault localization

**DOI:** 10.7717/peerj-cs.1071

**Published:** 2022-09-07

**Authors:** Heling Cao, Fei Wang, Miaolei Deng, Lei Li

**Affiliations:** 1 College of Information Science and Engineering, Henan University of Technology, ZhengZhou, HeNan, China; 2 Henan International Joint Laboratory of Grain Information Processing, Henan University of Technology, ZhengZhou, HeNan, China; 3 Key Laboratory of Grain Information Processing and Control, Ministry of Education, ZhengZhou, HeNan, China

**Keywords:** Software debugging, Fault localization, Dynamic slicing, Debugging cost

## Abstract

**Background:**

Spectrum-based Fault localization have proven to be useful in the process of software testing and debugging. However, how to improve the effectiveness of software fault localization has always been a research hot spot in the field of software engineering. Dynamic slicing can extract program dependencies under certain conditions. Thus, this technology is expected to benefit for locating fault.

**Methods:**

We propose an improved dynamic slicing for spectrum-based fault localization under a general framework. We first obtain the dynamic slice of program execution. Secondly, we construct a mixed slice spectrum matrix from the dynamic slice of each test case and the corresponding test results. Finally, we compute the suspiciousness value of each statement in the mixed-slice spectram matrix.

**Results:**

To verify the performance of our method, we conduct an empirical study on 15 widely used open-source programs. Experimental results show that our approach achieves significant improvement than the compared techniques.

**Conclusions:**

Our approach can reduce approximately 1% to 17.79% of the average cost of code examined significantly.

## Introduction

With the development of the software, locating fault is becoming more and more challenging. Fault localization is a part of software maintenance, which is estimated to cost 50*–*70% of program debugging (Jones & Harrold, 2005). Due to the expensive cost, the increase efficiency of fault localization can drastically decrease the cost of program debugging. Software fault localization mainly improve the efficiency through reducing the range of the search (Santelices et al., 2009; Agrawal & Horgan, 1990; Abreu, Zoeteweij & Van Gemund, 2006; Korel & Yalamanchili, 1994). Early the programmers set breakpoints by debugging tools to narrow down to search the range of the faults, but the manual debugging is low efficiency. Delta debugging techniques constantly exchange the memory state of success run and failure run to narrow the range of locating faults by the iteration of running programs (Santelices et al., 2009), but the cost of iterative search is larger. Dynamic slicing techniques removed non-related faulty statements to narrow the range of the search (Agrawal & Horgan, 1990). Soremekun et al. (2021) compared the effectiveness of spectrum fault localization against dynamic slicing. For single faults, dynamic slicing was eight percentage points more effective than the best performing spectrum fault localization; for 66% of the faults, dynamic slicing finds the fault earlier than the best performing spectrum fault localization. However, the calculation of dynamic slices less considers actual reference variables when the variable is defined. This leads that some irrelevant variable slices are contained among them, causing the results of slices is redundancy. To overcome this problem, we present an improved dynamic slicing approach based on defining variables influence set. When calculating the dynamic slicing, the current variables are found out when defining the actual reference, eliminating non-reference variables. Therefore, more precise dynamic slices are obtained.

Dynamic slicing technology narrows the scope of fault localization to a certain extent. However, the program after slicing is the statement set and the elements in the set are treated equally. The programmers need to check all statements in the dynamic slices to fault localization. In order to address this problem, we try to give the priority order of a slice element to reduce the statement number of fault localization being checked by programmers, and further improve the accuracy of fault localization. In this article, we present the fault localization using the improved dynamic slicing. First, we capture the dynamic slice information of program execution. Second, we construct a mixed slice spectrum matrix from the dynamic slice information of each test case execution and the corresponding test results. Finally, we compute the suspiciousness of entities in the mixed slice spectrum matrix to generate a fault localization report. Experimental results show that our method reduces almost 1% to 17.79% of the average cost of code examined significantly than the compared methods.

The main contributions of this article can be summarized as follows:

- We propose a novel approach using the improved dynamic slicing to spectrum-based fault localization.
- We improve dynamic slicing approach based on define variables influence set.

The rest of this article is organized as follows. “Background” presents the background on statistical fault localization and dynamic slicing. “Motivation” gives an example to show our approach. “Our approach” shows the framework and the exhibition of our approach. “Empirical study” describes our empirical study. “Related work” summarizes the related work of fault localization. Finally, “Conclusions and future work” concludes this article.

## Background

### Statistical fault localization

Statistical fault localization technology locates faults by calculating the suspiciousness of program statements. Jones & Harrold (2005) proposed the Tarantula technique, which computes the suspiciousness of statements and ranks them according to the suspiciousness. Similarly, Abreu, Zoeteweij & Van Gemund (2006) proposed another statistical fault localization technique, Ochiai, which finds the location of the fault by calculating the percentage of passing and failing tests of the executed statement. Specifically, fault program executes test cases. According to the result of the run, it further divides test cases into failed test cases and passed test cases. Finally, collecting the coverage information of test cases.

Suppose a program S = {s1, s2,…,sn} with n statements. The corresponding test suite T = {t1, t2,…,tm} consists of *m* test cases. After the test cases *T* are executed by the program *S*, we can obtain the program execution traces and the execution results. Finally, we use the execution traces and the execution results conveniently to get a program coverage matrix of *m* × *n*. There are a lot of approaches to statistical fault localization using the calculation formula of suspiciousness, among which the representative ones are Tarantula (Jones, Harrold & Stasko, 2002), Ochiai (Abreu, Zoeteweij & Van Gemund, 2007), *etc*.

Although there are great differences in the form of SBFL methods, the specific construction of SBFL methods is based on some common assumptions (Steimann, Frenkel & Abreu, 2013).

- Faulty statements may be covered by failing test cases and may be covered by passing test cases.
- Every failed test case executes at least one fault whose execution causes the failure.
- The distribution of faults in a program cannot be predicted. The prior probability distribution of faultiness is unknown.
- For the purpose of measuring the performance of a fault locator, we assume that upon inspection, a programmer always recognizes a faulty statement as such.

Xie et al. (2013) summarized additional assumptions when they theoretically analyzed the fault localization method:

- The fault localization method knows in advance how the test cases will perform in the program.
- The test case will always produce the same running result on the program regardless of the environment settings.
- The defective program can be 100% covered by the selected set of test cases, and contains at least one pass test case and one failing test case available.

The research and analysis of fault localization in this article are also based on the above assumptions.

### Program slicing

Program slicing is an important program analysis and understanding technology, which mainly analyze the data dependencies and control dependencies of the program to obtain the relevant features inside the program (Korel & Yalamanchili, 1994). Then understanding the entire program by analyzing the sliced program. At the same time, program slicing can be used to remove statements unrelated to program faults to narrow the scope of bug search, thereby reducing the cost of fault localization. Program slicing is widely used in software fault localization research.

**Control Flow Graph:** Control Flow Graph (CFG) is a directed graph, represented by CFG (N, E, Entry, Exit), where *N* is the set of nodes, representing program statements; *E* is a set of edges that represent the control flow relationship between nodes; Entry and Exit represent the entry and exit nodes of the program, respectively.

**Program Dependence Graph:** Program Dependence Graph (PDG) is a directed graph formed by removing the control flow edge from the control flow graph CFG and adding the control dependency and data dependency edges, which is expressed in the form of a two-tuple PDG (S, E). Where *S* is the node set of CFG, *E* is the set of edges, and edges represent the data dependency or control dependency between two nodes.

**System Dependence Graph:** System Dependence Graph (SDG), SDG = {GPDGs, EInterpro} of multi-process program *P* is a directed graph. Where GPDGs is the PDG set, and each process is represented as a PDG; EInterpro is the set of inter-procedure calling edges and dependent edges. Data-dependent edges represent data flow between actual and formal parameters.

**The Static Slicing:** Given a slicing criterion $C =\lt s, V \gt$, where *s* is the interesting points in the program and *V* is the set of variables. A static slice of a program *P* is the set of all statements affecting the set of variables *V* in a point of interest *s* (a statement or block of statements).

**The Dynamic Slicing:** Given a slicing criterion $C =\lt I, s, V \gt$, where *I* is the input to the program, the *s* is the interest in the program, and *V* is the set of variables. Based on the input *I*, a dynamic slice is the set of all statements of the program *P* that affect the variable set *V* in the point of interest *s* (a statement or block of statements) in this execution.

The concept of program slicing was first proposed by Weiser in his doctoral dissertation in 1979 (Weiser, 1979). Subsequently, Weiser (1984) proposed a program slicing algorithm based on the control flow graph equation by analyzing the data flow in the program, which provided theoretical support for the development of program slicing technology. Subsequently, many researchers began to pay attention to the study of program slicing technology, and achieved some important research results. The researchers found it difficult to establish data flow equations for multiple processes using Weiser’s method.

To solve this issue, Ottenstein & Ottenstein (1984) mapped the program statements to the nodes of the directed graph when analyzing the dependencies between the program statements, and added the edges of the directed graph if there were dependencies between the statements, and finally abstracted the source program. is the program dependency graph. Ferrante, Ottenstein & Warren (1987) implemented single-process program slicing and proposed a graph reach ability slicing algorithm that uses program dependency graphs to represent programs. Horwitz, Reps & Binkley (1990) implemented program slicing for multiple processes on the basis of (Ferrante, Ottenstein & Warren, 1987), and proposed a two-stage graph reach ability slicing algorithm using a system dependency graph.

At present, the typical program slicing algorithm is based on the data flow equation and the graph reach ability algorithm, and other algorithms are extended or improved from these two types of algorithms. The following briefly introduces the program slicing algorithm based on data flow and the program slicing algorithm based on graph reach ability.

- Dataflow-based program slicing algorithm. The basic idea is to first calculate the control dependencies in the program, and then according to the data flow transfer in the control flow analysis program, iteratively construct slices by calculating the statements of the direct and indirect dependencies of the variables. The first program slice proposed by Weiser (1982) belongs to static backward slice, which is obtained based on the control flow graph and through two-layer iterative data flow analysis. The first layer calculates data dependencies, and calculates the variables that directly and indirectly depend on the variables in the slicing criterion; the second layer calculates control dependencies, by tracking the transferable data flow, calculating its control dependent statements, iteratively analyzing the newly obtained statement set; finally, the desired slice is obtained.
- Graphreach-based program slicing algorithm. The basic idea is to obtain program slices by traversing the constructed Program Dependency Graph (PDG) or System Dependency Graph (SDG), and finding other nodes that the program can reach from the slicing criterion according to the dependencies. For example, Ottenstein & Ottenstein (1984) solved intra-procedural slices using a graph reach ability algorithm with the help of a constructed program dependency graph. Horwitz, Reps & Binkley (1990) constructed a system dependency graph, and also implemented program slicing between procedures on SDG using a graph reach ability algorithm.

By program slicing, all statements can be classified into correct set of statements and suspicious set of statements. Therefore, most of the slicing-based fault location techniques use different slices (Zhang, Gupta & Zhang, 2004), execution slices (Xu et al., 2011) to locate faults in order to minimize the scope of suspicious statements. Singh & Mohapatra (2018) presented a context-sensitive dynamic slicing technique for concurrent programs. To effectively represent the concurrent aspect-oriented programs, Singh & Mohapatra (2018) proposed an intermediate graph called the multithreaded aspect-oriented dependence graph. Li & Orso (2020) presented a technique that computes memory-address dependence and represents them on standard dynamic dependence graphs for better supporting software debugging. Zhang (2021) attempted to study a light-weight approach of static program slicing, which works as a dataflow analysis on low-level virtual machine.

## Motivation

**Figure 1:**
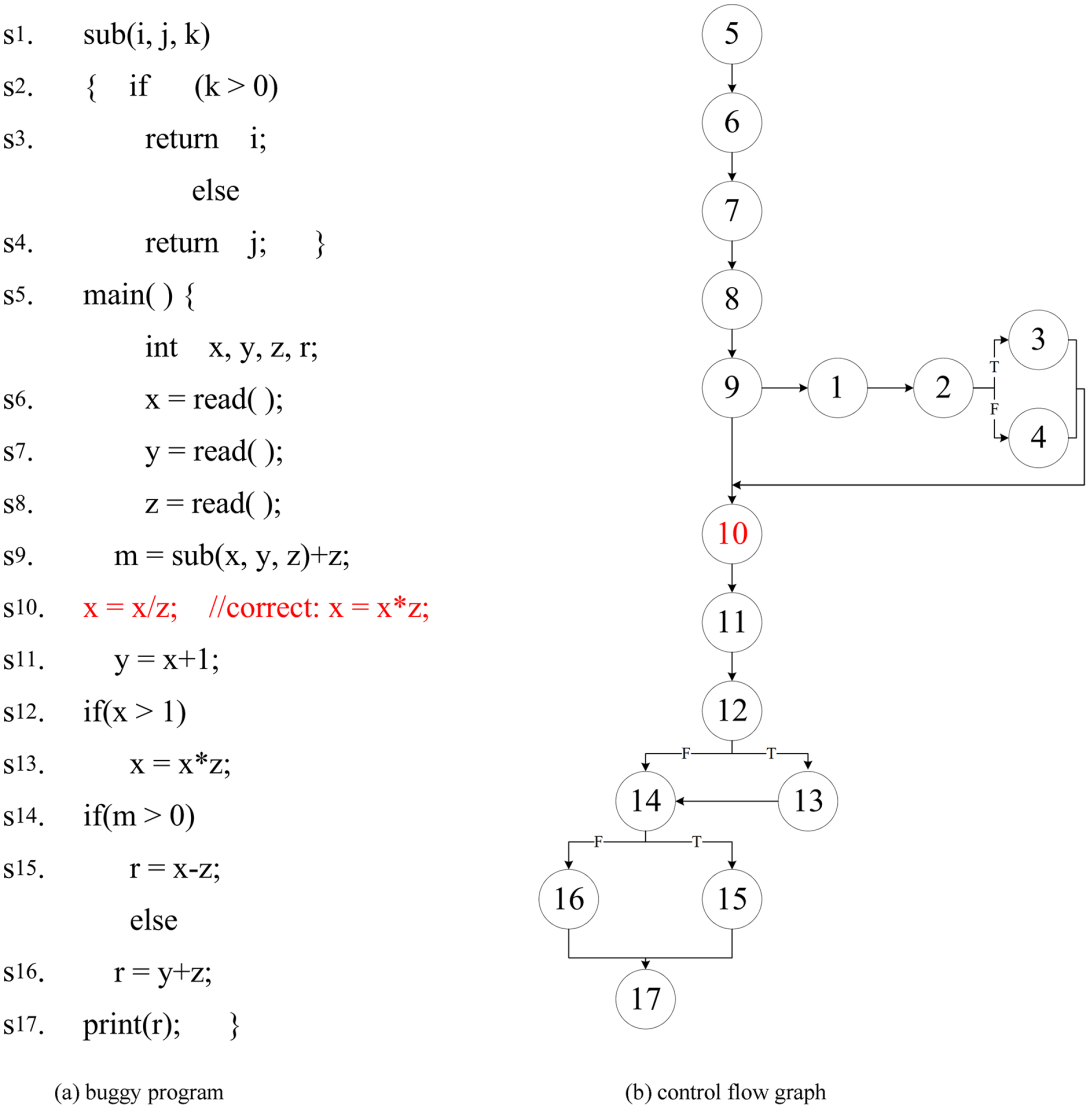
This is a buggy program and it’s control flow graph.

Software fault localization has a problem that the search domain is too large and the accuracy is not high. Fault localization approaches often use dynamic slicing techniques to narrow down the scope of the fault. However, the existing forward computing dynamic slicing approaches (Zhang, Gupta & Zhang, 2004; Xu et al., 2011) calculate the slicing of variables defined by the statement. These approaches take into account the slicing of all referenced variables in the statement, rather than the slicing of the defined variable. Which results in the redundancy of slicing results. For this reason, this article analyzes the impact set of the defined variables, and considers the slice results of the actual reference variables when the variables are defined, thus improving the original dynamic slicing approach (Xu et al., 2011). The dynamic slicing technology reduces the scope of fault localization to a certain extent, but the post-slice program is a collection of statements, and the elements in the collection have no order. The tester needs to check all the statements in the slice to locate the fault. For this purpose, our approach combined with the proposed association analysis and sorting strategy is used to determine the priority order of statements checking, which is used to improve the accuracy of the fault localization. The motivation for this approach is illustrated by the following program in Fig. 1A (The fault is in ${s_{10}}$). The program of control flow graph follow as Fig. 1B.

For the example program in Fig. 1, we design the following eight test cases: ${t_1}$(5,2,5), ${t_2}$(4,1,4), ${t_3}$(−4,2,−2), ${t_4}$(1,4,1 ), ${t_5}$(1,0,−1), ${t_6}$(−2,-4,1), ${t_7}$(0,4,1) and ${t_8}$(0,0,−1). We collect the coverage information of their execution respectively, (original/improved) dynamic slice information ( $\lt$ I, ${s_{17}}$, r $\gt$ is the slice criterion). The fault localization effect of the Tarantula (Jones & Harrold, 2005; Jones, Harrold & Stasko, 2002) approach on coverage information, original dynamic slice (Xu et al., 2011) and improved dynamic slice is shown in Table 1 (“•” indicates that the statement is covered, and “○” indicates that the statement is not covered). Column 1 is the statement number, and column 2 lists the override information and the statement sort result calculated on the Tarantula. The fault program is checked by the rank from small to large. Column 3 is the original dynamic slice and the result of the statement on which Tarantula is calculated. Column 4 is the improved dynamic slice and the result of the statement on which Tarantula is calculated. T/F at the second line from the bottom indicates that the program execution result is true (T) or failed (F). The last line “Fault. Rank” indicates that the two approaches locate the number of statements that need to be checked for faults. For example, “4–14” means that four statements is examined to locate fault at the best case, and 14 statements is examined to locate fault at the worst case.

**Table 1:** Ranking results between original dynamic slicing and improved dynamic slicing.

Statement	Original dynamic slice									Improved dynamic slice								
	t1	t2	t3	t4	t5	t6	t7	t8	Tarantula	t1	t2	t3	t4	t5	t6	t7	t8	Tarantula
s1	∙	∙	∙	∙	∙	∙	∙	∙	3	∙	∙	∙	∙	∙	∙	∙	∙	3
s2	∙	∙	∙	∙	∙	∙	∙	∙	3	∙	∙	∙	∙	∙	∙	∙	∙	3
s3	∙	∙	∘	∙	∘	∙	∙	∘	2	∙	∙	∘	∙	∘	∙	∙	∘	2
s4	∘	∘	∙	∘	∙	∘	∘	∙	11	∘	∘	∙	∘	∙	∘	∘	∙	10
s6	∙	∙	∙	∙	∙	∙	∙	∙	3	∙	∙	∙	∙	∙	∙	∙	∙	3
s7	∙	∙	∙	∙	∙	∙	∙	∙	3	∘	∘	∙	∘	∙	∘	∘	∙	10
s8	∙	∙	∙	∙	∙	∙	∙	∙	3	∙	∙	∙	∙	∙	∙	∙	∙	3
s9	∙	∙	∙	∙	∙	∙	∙	∙	3	∙	∙	∙	∙	∙	∙	∙	∙	3
s10(f)	∙	∙	∙	∙	∙	∙	∙	∙	3	∙	∙	∙	∙	∙	∙	∙	∙	3
s11	∘	∘	∙	∘	∙	∙	∘	∙	12	∘	∘	∙	∘	∙	∙	∘	∙	12
s12	∘	∘	∘	∘	∘	∘	∘	∘		∘	∘	∘	∘	∘	∘	∘	∘	
s13	∘	∘	∘	∘	∘	∘	∘	∘		∘	∘	∘	∘	∘	∘	∘	∘	
s14	∙	∙	∙	∙	∙	∙	∙	∙	3	∙	∙	∙	∙	∙	∙	∙	∙	3
s15	∙	∙	∘	∙	∘	∘	∙	∘	1	∙	∙	∘	∙	∘	∘	∙	∘	1
s16	∘	∘	∙	∘	∙	∙	∘	∙	12	∘	∘	∙	∘	∙	∙	∘	∙	12
s17	∘	∘	∘	∘	∘	∘	∘	∘		∘	∘	∘	∘	∘	∘	∘	∘	
T/F	F	F	F	T	T	T	T	T		F	F	F	T	T	T	T	T	
Fault.Rank									3–10									3–9

However, the existing forward-calculated dynamic slice side (Zhang, Gupta & Zhang, 2004; Xu et al., 2011) calculates the dynamic slice of the variable m defined in the statement ${s_9}$, and contains the dynamic slice of the reference variable y, and the result is redundant. The dynamic slice obtained by the approach in this study is more accurate, which makes the fault localization more accurate.

As can be seen from columns 2 and 3 of Table 1, the original dynamic slice is smaller than the scale of coverage information. For example, when t1 executes, the dynamic slice removes irrelevant statements ${s_{11}}$, ${s_{12}}$ and ${s_{17}}$. The Tarantula approach locates faults on the original dynamic slice and improved dynamic slice, and the number of statements to check is “3–10” and “3–9”, respectively. It can be seen that dynamic slicing improves the accuracy of fault localization. As can be seen from the third and fourth columns, the improved dynamic slice is smaller than the original dynamic slice size. For example, when ${t_1}$ is executed, the improved dynamic slice removes the irrelevant statement ${s_7}$. The reason for this is that the actual reference variable of the defined variable is considered, and the slice of the unrelated variable y is removed to reduce the slice size. The Tarantula approach needs to check “3–9” statements to locate faults on the improved dynamic slice, which shows that the improved dynamic slice further improves the accuracy of fault localization compared with the original dynamic slice.

## Our approach

**Figure 2:**
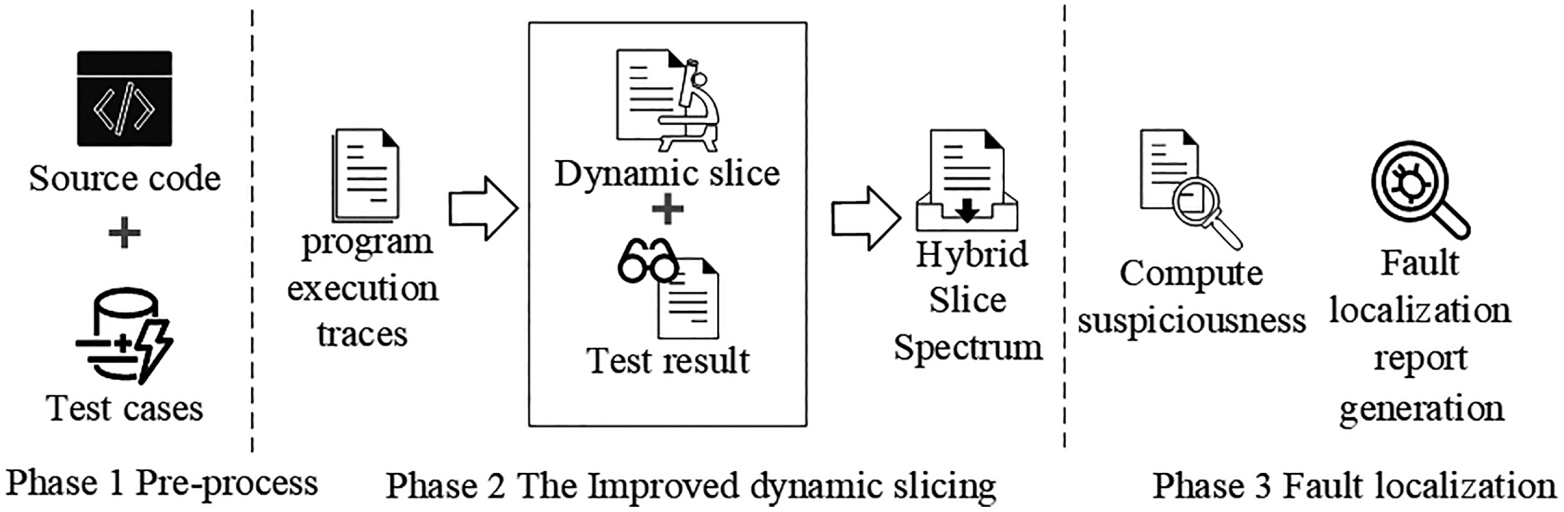
The framework of our approach.

In this section, we present an improved dynamic slicing for fault localization. Our approach collects the dynamic slice information of program execution. Next, we construct a mixed slice spectrum matrix from the dynamic slice information of each test case execution and the corresponding test results. Finally, we compute the suspiciousness ranking of each statement in the mixed slice spectrum matrix. The framework of our approach is shown in Fig. 2.

### The improved dynamic slicing

Dynamic slicing is an effective debugging technique, which has been widely used in fault localization. When program execution results in a fault output or exception, the debugger usually wants to quickly ascertain the range of possible fault statements. By calculating the backward dynamic slice of the fault output statement, the dynamic slice can narrow the range of fault localization. Compared with the backward calculation, after the occurrence of the fault, the forward approach can generate a dynamic slice, which is according to the direct dynamic dependency of bug output statement. The forward approach does not need to traverse the dynamic dependency in the back, so it can calculate the dynamic slice result more quickly when a program runs incorrectly.

Dynamic slicing technology (Korel & Yalamanchili, 1994) reduces the fault search scope by removing statements that are not related to program faults. However, less consideration is given to variables actually referenced in variable definition during dynamic slice calculation, which results in the inclusion of slices of some unrelated variables and the redundancy of slice results. Therefore, we propose a dynamic slicing approach, which is based on the defined variable influence set. When calculating dynamic slicing, this approach finds out the variables actually referenced by the current variable at the time of definition and excludes the unreferenced variables, in order to get more accurate dynamic slicing.

Therefore, the forward calculation approach is used to calculate the program’s dynamic slicing, and the influence set analysis of the defined variables is carried out to improve the slicing accuracy, and the obtained dynamic slicing results are used for fault localization. The forward calculation method obtains the dynamic slice of interest points in the program through the direct dynamic dependencies of the interest points in the program. This method needs to maintain the slice results of each execution instance of the program (Korel & Yalamanchili, 1994; Zhang, Gupta & Zhang, 2004). The forward calculation method can generate a dynamic slice of the fault output statement according to the direct dynamic dependency of the fault output statement after an error occurs, and does not need to traverse the dynamic dependency backward. When the program fails so the dynamic slice result can be calculated more quickly. The following are the relevant definitions.

**Definition1.** Influence set *Influence*[*v*]. For a given statement *s*, the variable $v \in De\;f[s]$, the influence set *Influence*[*v*]= {*v′*| The *v′ is the variabl e actually referenced when v is defined*, $v^{\prime}\in U \;se[s]$}. Where, the definition set *Def*[*s*] is the set of variables defined in statement *s*, and the reference set *Use*[*s*] is the set of variables referenced in statement *s*.

In this section, the influence set analysis is performed on the defined variables to remove irrelevant variables in order to reduce the redundancy of the slicing results.

**Definition2.** Hybrid Slicing Spectrum Matrix (HSSM). For a program *P* consisting of *n* statements *s* and a given m test cases, each execution of the test case yields a dynamic slice (*slice*) and the corresponding execution result. The mixed slice spectrum matrix is a two-dimensional matrix composed of multiple dynamic slices and execution results.

(1) $${\rm HSSM} = \left[ {{{\rm M}_{{\rm m} \times {\rm n}}}{{\rm N}_{{\rm m} \times 1}}} \right]\quad \forall {{\rm a}_{{\rm ij}}} \in {{\rm M}_{{\rm m} \times {\rm n}}},\forall {{\rm b}_{{\rm ij}}} \in {{\rm N}_{{\rm m} \times 1}}$$

(2) $${{\rm a}_{{\rm ij}}} = \left\{ {\matrix{ 1 \hfill & {\; {\rm s} \in {\rm slice }} \hfill \cr 0 \hfill & {\; {\rm s}\ \notin\ {\rm slice }} \hfill \cr } \quad 1 \le {\rm i} \le {\rm m},1 \le {\rm j} \le {\rm n}} \right.$$

(3) $${b_{ij}} = \left\{ {\matrix{ {\rm T} & {{\rm true }} \cr F & {{\rm failed }} \cr } \quad 1 \le i \le m,j = n + 1} \right.$$

This hybrid slice spectrum matrix uses the program dynamic slice information to extend the program coverage information and combines it with the corresponding program execution results to generate the hybrid slice spectrum matrix.

Program slicing can extract a set of statements from a program according to a certain slicing criterion, we use the fault-related criteria of failed runs by program slicing to collect the slices. Our approach computes the slices according to the Program Dependence Graph (PDG) and the slice of statements with a forward computing algorithm. Algorithm 1 describes the forward computing dynamic slicing algorithm based on the influence set of defined variables. Algorithm 1 takes as input the current execution instance ${s^i}$, current execution method call statement (*call*), dynamic control dependency of ${s^i}$ ( $dynCD\left[ {{s^i}} \right]$), dynamic data dependence of ${s^i}$ ( $dynDD\left[ {{s^i}} \right]$) and slice of predicate pre (*slicePred*). The final output is the statement *s* refers to the dynamic slice of the variable use and the statement *s* defines the dynamic slice of the variable *def*. Lines 1–3 calculate the dynamic *slice*[*use*] of the variable use in the reference set *Use*[*s*] of statement *s*. $dynDD\left[ {{s^i}} \right]$ obtains a dynamic *slice*[*use*] of the referenced variable based on the dynamic data dependency of the current execution instance ${s^i}$. Lines 4–10 calculate the influence set of the defined variable *def* according to definition 1: *Influence*[*def*]. Lines 11–18 compute the dynamic *slice*[*def*] of the variable def in the definition set of *Def*[*s*]. Line 11 executes the dynamic control dependency $dynCD\left[ {{s^i}} \right]$ of the instance si to get the *slicePred* of the control dependent predicate *pred*. Lines 12–18 calculate the dynamic slice *slice*[*def*] of the defined variable, which is consist of four parts: the current execution statement *s*, the call statement call, the control dependent predicate slice *slicePred*, and the slice of the variables influence in the defined variable influence set *Influence*[*def*].

**Algorithm 1:** Dynamic slicing algorithm based on the influence set of defined variables.

**Require:** $s^{i}$ // Current execution instance
* call * // Current execution method call statement
* * $dynCD {[s^i]}$ // Dynamic control dependency of $s^{i}$
* $dynDD {[s^i]}$* // Dynamic data dependency of $s^{i}$
* slicePred* // Slice of predicate *Pred*
**Ensure:** *slice*[*use*] // The statement *s* refers to the dynamic slice of the variable *use*
* slice*[*def* ] // Statement *s* defines the dynamic slice of the variable *def*
1: **for** each use $\in$ *Use*[*s*] **do**
2: find *slice*[*use*] by $dynDD {[s^i]}$;
3: **end for**
4: **for** each def $\in$ *Def* [*s*] **do**
5: *Influence*[*def* ] = {};
6: **for** each use $\in$ *Use*[*s*] **do**
7: **if** use affect the value of *def*
8: **then** add *use* to *Influence*[*def*]
9: **end for**
10: **end for**
11: find *slicePred* by $dynCD {[s^i]}$;
12: **for** each *def* ∈ *Def* [*s*] **do**
13: *slice_T* = { s, call } $\cup$ *slicePred*;
14: **for** each *influence* ∈ *Influence*[*def*] **do**
15: *slice_T* = { s, call } $\cup$ *slice*[*influence*];
16: **end for**
17: *slice*[*def* ] = *slice_T*;
18: **end for**

### Fault localization

Dynamic slicing of programs can narrow the search for software fault localization by obtaining statements related to the slicing criterion. Then, we compute the suspiciousness of entities in the dynamic slice by the formulas of fault localization to generate a fault localization report. For passed tests and failed tests, more uncovered entities with passing executions and covered entities with failed executions should be assigned a higher degree of suspiciousness. Moreover, the covered entities by more passed executions and the uncovered entities by the failed executions should be assigned a lower suspiciousness. Therefore, the number of covered entities should have more weight than the number of uncovered entities in the suspiciousness calculation. Over the past few decades, dozens of doubtful formulas have been proposed and evaluated through empirical research. In our approach, we apply Tarantula (Jones & Harrold, 2005; Jones, Harrold & Stasko, 2002), Naish1 (Naish, Lee & Ramamohanarao, 2011), GP02 (Yoo, 2012), Ochiai (Abreu, Zoeteweij & Van Gemund, 2007), Jaccard (Chen et al., 2002) and GP13 (Yoo, 2012) to generate statement checking ranking based on coverage information. The suspiciousness value formulas of Tarantula, Naish1, GP02, Ochiai, Jaccard and GP13 as follows.

(4) $$Tarantula ({\rm s}) = \displaystyle{{{n_{ef}}(s)/{n_f}} \over {{n_{ef}}(s)/{n_f} + {n_{ep}}(s)/{n_p}}}$$

(5) $$Naish 1({\rm s}) = \left\{ {\matrix{ { - 1} & {{n_{ef}}(s) \lt {n_f}} \cr {{n_p} - {n_{ep}}(s)} & {{n_{ef}}(s) = {n_f}} \cr } } \right.$$

(6) $$\left. {GP 02({\rm s}) = 2\left( {{{\rm n}_{{\rm ef}}}({\rm s}) + \sqrt {{{\rm n}_{\rm p}}} + \sqrt {{{\rm n}_{{\rm ep}}}({\rm s}} } \right)} \right)$$

(7) $$Ochiai ({\rm s}) = \displaystyle{{{n_{ef}}(s)} \over {\sqrt {{n_f} \times \left( {{n_{ef}}(s) + {n_{ep}}(s)} \right)} }}$$

(8) $$Jaccard ({\rm s}) = \displaystyle{{{n_{ef}}(s)} \over {{n_f} + {n_{ep}}(s)}}$$

(9) $$GP 13({\rm s}) = {n_{ef}}(s) \times \left( {1 + \displaystyle{1 \over {2{n_{ef}}(s) + 2{n_{ep}}(s)}}} \right)$$

where, ${n_f}$ and ${n_p}$ denote the number of failed and passed test cases respectively. ${n_{nf}}(s)$ and ${n_{np}}(s)$ denote the number of failed and passed test cases that are not covered entities. ${n_{ef}}(s)$ and ${n_{ep}}(s)$ denote the number of failed and passed test cases that are covered entities. The coverage information of the entity, we can get the corresponding suspiciousness of the entity through the suspiciousness formula.

## Empirical study

To verify the effectiveness of our approach, we compare our approach with both the original dynamic slice and ADBS on the fault localization. Lyle (1985) proposed the debug based on program slicing. The data and/or control dependencies of program entities have been obtained by program slicing, which has been used to improve the effectiveness of fault localization. The approximate dynamic backward slicing (ADBS) approach has been presented by Lei et al. (2012). The ADBS approach computes the statements in the intersection of the static backward slice and the set of execution slices to improve the effectiveness of fault localization. In this experiment, we employ six formulas (*i.e*., Tarantula, Naish1, GP02, Ochiai, Jaccard, and GP13) and compare our approach and other approaches using the same evaluation formula, respectively. For example, Tarantula is applied to the original dynamic slice for locating faults, which is denoted as D-Tarantula; Tarantula is applied to the improved dynamic slice to locate faults, which is denoted as DS-Tarantula. Whereas, Tarantula is applied to ADBS to locate faults, which is denoted as ADBS-Tarantula.

### Experiment subjects

In this Section, 15 Java programs are selected as the benchmark for testing, and the detailed description is shown in Table 2. Where columns 1–3 are the program name, description and lines of code (excluding blank lines and comment lines), and columns 4 and 5 are the number of fault versions and test cases used in the experiment. The first six programs in the table are the Siemens Test Suite. Siemens Suite (except jtcas) is a program that converts a C version to a Java version by Santelices et al. Jtcas, Nanoxml, and XML-security with the corresponding test cases are all derived from SIR (Do, Elbaum & Rothermel, 2005). The latter three projects JFreeChar, Joda-Time and Mockito are from the Defects4J library (Just, Jalali & Ernst, 2014). The numbers of executable lines of all the subjects are ranging from hundreds of lines to tens of thousands of lines. This allows us to evaluate across a very broad spectrum of programs and allows us to have more confidence in the ability to generalize our results.

**Table 2:** Subject programs.

Procedure	Description	Lines of code	Number of versions	Number of test cases	Fault type
jtcas	Collision avoidance procedure	165	31	1,608	seeded
tot_info	Data statistics program	283	10	1,052	seeded
schedule	Priority scheduler	290	9	2,650	seeded
schedule2	Priority scheduler	317	8	2,710	seeded
print_tokens	Lexical analyzer	478	7	4,130	seeded
print_tokens2	Lexical analyzer	410	5	4,115	seeded
NanoXML v1	XML parser	3,497	7	214	real
NanoXML v2	XML parser	4,009	6	214	real
NanoXML v3	XML parser	4,608	8	216	real
NanoXML v5	XML parser	4,782	7	216	real
XML-sec v1	XML encryptor	21,613	7	92	real
XML-sec v2	XML encryptor	22,318	5	94	real
JFreeChart	Chart library	96,300	26	2,205	real
Joda-time	Time library	28,400	25	4,130	real
Mockito	Unit test framework	23,000	36	1,366	real

Our approach applied to single-bug version programs to locate fault. We removed two versions with no test case execution failure and eight versions with multiple faults from the 41 fault versions of jtcas, and selected the remaining 31 versions for testing. We randomly selected 10 of the 23 incorrect versions of the tot $\_$info program for testing, and selected nine fault versions of schedule. Selected 8 of the 10 fault versions of Schedule2 to test, removed the v4 version (fault is to remove the comment statement at 350 lines) and the v6 version with multiple faults. Test with seven fault versions of print $\_$tokens and five fault versions of print $\_$tokens2. In addition, a total of 28 fault versions of NanoXML and 12 fault versions of XML-security (abbreviated XML-sec) were selected for testing. In Defects4J, the part of the fault version of the program does not output a fault. We removed the v2 and v19 version of Time, and selected the remaining 25 versions for testing. We removed the v6 and v10 version of Mockito, and selected the remaining 36 versions for testing. Finally, we tested 197 single-fault versions of programs.

As shown in fourth column of Table 2, in the first six programs, all the faults were manually seeded by other researchers and the considered fault types include predicate based fault and assignment based fault. All creation faults are semantic bugs that do not incur crashes. These creation faults have been shown to simulate realistic faults well and provide reliable, trustworthy results. The latter nine projects have the real faults. Also packaged with each of these programs was a set of test cases and faulty versions. For all of our subject programs, any faulty versions that did not lead to at least one test case failure in our execution environment were excluded. In this way, we assure that each fault examined would be revealed by at least one test case.

### Evaluation metrics

In order to evaluate the effectiveness of various fault localization approaches, we use the following four criteria to evaluate the effectiveness from different perspectives.

**Cumulative Number of Statements Examined:** The number of cumulative examined statements for each program is the number of total statements that need to be checked when m faults are located in m fault versions. For m fault versions of any given program, M(i) and N(i) represent the number of statements that need to be checked when approach M and approach N locate a fault. If $\sum\limits_{{\rm i} = 1}^{\rm n} {\rm M} ({\rm i}) < \sum\limits_{{\rm i} = 1}^{\rm n} {\rm N} ({\rm i})$, it indicates that the approach M is more effective than the approach N.

**Cost Score:** Cost criterion is to evaluate the effectiveness of fault localization from the perspective of relative index, which was first proposed by Renieres & Reiss (2003). Cost is the ratio of the percentage of the number of statements (Rank of faults) that need be examined to the total number of all executable statements when finding errors in the program version in, as shown in Eq. (10).

(10) $${\rm cost } = \displaystyle{{{\rm Rank\; of\; faults }} \over {{\rm Number\; of \;executable\; statements }}} \times 100\%$$

Because the faulty statement may share the same suspiciousness with others, the programmer might need to examine only one of these statements fortunately (best cases) or to examine all these statements to locate the real fault unfortunately (worst cases) (Jones & Harrold, 2005). In the experiments, we obtain the average fault-localization costs per subject by the best fault-localization costs per subject and the worst fault-localization costs per subject. Finally, we use the average fault-localization costs as an evaluation indicator.

**EXAM Score:** To more intuitively compare the effectiveness of different approaches, we also use EXAM indicators for evaluation. The EXAM indicator is the ratio of the fault detection rate (*%* of faults located) to the code examined rate (*%* of code examined) (Renieres & Reiss, 2003), as shown in Eq. (11).

(11) $${\rm EXAM } = \displaystyle{{\% \;{\rm of \; fault \; located }} \over {\% \;{\rm of \;code \; examined }}}$$

In the early research, Renieres & Reiss (2003) used the test score EXAM to evaluate the efficiency of fault localization. Jones, Harrold & Stasko (2002) also used this benchmark to compare the efficiency of the fault localization approaches. In the effectiveness of the evaluation in locating fault, the higher the suspicious score is, the better effectiveness fault localization is.

**Recall at Top-N:** This dependent variable measures the number of faults with at least one faulty element within Top-N in the ranked list. This metric emphasizes earlier fault detection and has been widely used in fault localization work (Le, Oentaryo & Lo, 2015). Kochhar et al. (2016) reported that developers usually only inspect top-ranked program elements during fault localization. Therefore, following prior work, we use Top-N (N = 5, 10, 20, 30) in our experimental study.

## Data analysis

### Experiments using the cumulative number of statements examined

We can observe the cumulative number of statements examined between our technique and the original dynamic slice in Table 3. The improved dynamic slices contributed to the improvement of the accuracy in locating faults. From the comparison of Table 3, for each program, the cumulative number of statements examined by the improved dynamic slice is smaller than the original dynamic slice on five techniques (Tarantula, Nasish1, GP02, Ochiai, Jaccard, and GP13). For example, on the most popular Ochiai the number of examined statements by the improved dynamic slices to locate faults for jtcas program was reduced by 66 than the original dynamic slice, similarly, the number of examined statements for tot $\_$info programs was reduced by 197. In the last row of Table 3, Total Cumulatives, the total number of statements for the overall topic that each method needs to examine is stated. We can also observe that DS-Naish1 performs best and has only 85,534 total check statements, which is much less expensive than all the comparison techniques over 15,000 lines.

**Table 3:** The cumulative number of statements examined between improved and original dynamic slicing.

Subject	Tarantula			Naish1			GP02			Ochiai			Jaccard			GP13		
	D	DS	ADBS	D	DS	ADBS	D	DS	ADBS	D	DS	ADBS	D	DS	ADBS	D	DS	ADBS
jtcas	555	490	538	483	382	423	550	425	467	526	460	501	531	465	505	552	446	486
tot_info	450	260	323	450	276	349	480	280	363	399	202	295	407	208	291	499	279	362
schedule	165	150	205	145	140	295	200	162	318	136	119	274	222	205	359	203	173	327
schedule2	597	159	321	501	210	272	525	216	288	601	165	227	651	222	274	538	221	273
print_tokens	404	182	195	289	159	176	320	129	143	352	133	146	316	126	138	331	143	155
print_tokens2	400	211	395	421	216	405	408	184	378	376	184	358	212	158	342	419	192	366
NanoXML v1	1,560	1,350	1,670	1,250	1,101	1,491	1,501	1,208	1,529	1,473	1,269	1,579	1,527	1,323	1,634	1,512	1,309	1,619
NanoXML v2	990	820	1,247	810	762	1,139	880	792	1,229	898	729	1,166	888	719	1,166	881	802	1,239
NanoXML v3	2,221	1,906	1,990	1,561	1,401	1,475	1,800	1,445	1,539	2,002	1,687	1,770	1,641	1,425	1,509	1,811	1,603	1,686
NanoXML v5	2,632	2,100	2,358	2,128	1,629	1,867	2,100	1,922	2,181	2,473	1,946	2,194	2,353	1,826	2,084	2,104	2,006	2,254
XML-sec v1	3,101	2,200	2,539	2,601	2,400	2,739	2,100	1,901	2,250	2,914	2,015	2,353	2,752	1,953	2,292	2,103	2,038	2,376
XML-sec v2	2,150	1,702	2,055	2,201	1,938	2,292	2,108	2,015	2,368	2,057	1,609	1,952	1,974	1,626	1,979	2,112	2,010	2,353
JFreeChart	43,622	43,219	44,497	42,094	41,903	41,869	42,085	41,954	42,013	42,816	42,413	43,497	43,061	42,567	42,161	42,107	42,065	42,095
Joda-Time	25,156	24,934	24,952	23,052	22,831	22,930	25,114	24,959	24,851	24,363	24,143	24,063	25,024	24,917	24,524	25,139	25,060	25,117
Mockito	11,236	10,970	11,141	10,373	10,186	10,247	11,001	10,843	15,673	11,007	10,736	11,001	10,391	10,220	10,410	11,092	10,954	11,461
Cumulative	95,239	90,653	94,426	88,359	85,534	87,969	91,172	88,435	95,590	92,393	87,810	91,376	91,950	87,960	89,668	91,403	89,301	92,169

Furthermore, we can observe the cumulative number of statements examined between our technique and ADBS in Table 3. The improved dynamic slices better than ADBS in locating faults. From the comparison of Table 3, for each program, the cumulative number of statements examined by the improved dynamic slice is smaller than ADBS on six techniques (Tarantula, Nasish1, GP02, Ochiai, Jaccard, and GP13). For example, on the most popular Ochiai the number of examined statements by the improved dynamic slices to locate faults for jtcas program was reduced by 41 than ADBS, similarly, the number of examined statements for tot $\_$info programs was reduced by 93. In the last row of Table 3, Total Cumulatives, we can also observe that our approach performs better than ADBS in all formula. Therefore, it is clear that our proposed technique improves the effectiveness of fault localization.

### Experiments using the average cost metric

As shown in Table 4, we compare the average cost of fault localization our method and the original dynamic slice on each topic. From Table 4, we can observe that for all subjects, the average cost of fault localization of our method is much smaller than that of the original dynamic slice. The last row of Table 4 illustrates the average cost across all topics for each technique. It shows that, for all subjects, the average cost of our improved dynamic slice is less than that of original dynamic slice on Tarantula, Naish1, GP02, Ochiai, Jaccard and GP13 which are 5.09*%*, 4.79*%*, 4.87*%*, 4.53*%*, 4.76*%* and 5.08*%*, respectively. Furthermore, the cost of DS-Tarantula is much smaller than that of D-Tarantula on schedule2, which is 6.27*%*, whereas the cost of DS-GP02 is much smaller than that of D-GP02 on schedule2, which is 8.52*%*.

**Table 4:** Average cost comparison between improved and original dynamic slicing.

Subject	Tarantula			Naish1			GP02			Ochiai			Jaccard			GP13		
	D (%)	DS (%)	ADBS (%)	D (%)	DS (%)	ADBS (%)	D (%)	DS (%)	ADBS (%)	D (%)	DS (%)	ADBS (%)	D (%)	DS (%)	ADBS (%)	D (%)	DS (%)	ADBS (%)
jtcas	10.85	9.58	10.52	9.44	7.47	8.27	10.75	8.31	9.13	10.28	8.99	9.79	10.38	9.09	9.87	10.79	8.72	9.50
tot_info	15.90	9.19	11.41	15.90	9.75	12.33	16.96	9.89	12.83	14.10	7.14	10.42	14.38	7.35	10.28	17.63	9.86	12.79
schedule	6.32	5.75	7.85	5.56	5.36	11.30	7.66	6.21	12.18	5.21	4.56	10.50	8.51	7.85	13.75	7.78	6.63	12.53
schedule2	23.54	6.27	12.66	19.76	8.28	10.73	20.70	8.52	11.36	23.70	6.51	8.95	25.67	8.75	10.80	21.21	8.71	10.76
print_tokens	12.07	5.44	5.83	8.64	4.75	5.26	9.56	3.86	4.27	10.50	3.97	4.36	9.44	2.36	4.12	9.89	4.27	4.63
print_tokens2	19.51	10.29	19.27	20.54	10.54	19.76	19.90	8.98	18.44	18.34	8.98	17.46	10.34	7.31	16.68	2.04	9.37	17.85
NanoXML v1	6.37	5.51	6.82	5.11	4.50	6.09	6.13	4.93	6.25	6.02	5.18	6.45	6.24	5.42	6.68	6.18	5.35	6.61
NanoXML v2	4.12	3.41	5.18	3.37	3.17	4.74	3.66	3.29	5.11	3.73	3.03	4.85	3.69	3.16	4.85	3.66	3.33	5.15
NanoXML v3	6.02	5.17	5.40	4.23	3.80	4.00	4.88	3.92	4.17	5.43	4.58	4.80	4.45	4.32	4.09	4.91	4.35	4.57
NanoXML v5	7.86	6.27	7.04	6.36	4.87	5.58	6.27	5.74	6.52	7.39	5.81	6.55	7.03	5.80	6.23	6.29	5.99	6.73
XML-sec v1	2.05	1.45	1.68	1.72	1.59	1.81	1.39	1.26	1.49	1.93	1.33	1.56	1.82	1.35	1.51	1.39	1.34	1.57
XML-sec v2	1.80	1.43	1.84	1.84	1.62	2.05	1.77	1.69	2.12	1.84	1.44	1.75	1.77	1.30	1.77	1.89	1.80	2.11
JFreeChart	1.74	1.73	1.78	1.68	1.67	1.67	1.68	1.67	1.68	1.71	1.17	1.74	1.72	1.92	1.68	1.68	1.68	1.68
Joda-Time	3.54	3.51	3.51	3.25	3.21	3.23	3.54	3.52	3.50	3.43	3.40	3.39	3.52	3.28	3.45	3.54	3.53	3.54
Mockito	1.36	1.32	1.35	1.25	1.23	1.24	1.33	1.31	1.89	1.32	1.30	1.33	1.25	1.29	1.26	1.34	1.32	1.38
Average cost	8.20	5.09	6.81	7.24	4.79	6.54	7.75	4.87	6.73	7.66	4.53	6.26	7.35	4.76	6.47	7.91	5.08	6.76

Than, we compare the average cost of fault localization our method and ADBS on each topic, as shown in Table 4. The last row of Table illustrates the average cost across all topics for each technique. From Table 4, we can observe that for all subjects, the average cost of fault localization of our method is smaller than that of ADBS. Furthermore, the cost of DS-Tarantula is smaller than that of ADBS-Tarantula on JFreeChart, which is 1.73%, whereas the cost of DS-Ochiai is smaller than that of ADBS-Ochiai on JFreeChart, which is 1.17%. That is to say, our approach is actually more effective than the original dynamic slice and ADBS in the experiments.

### Experiments using EXAM metric

**Figure 3:**
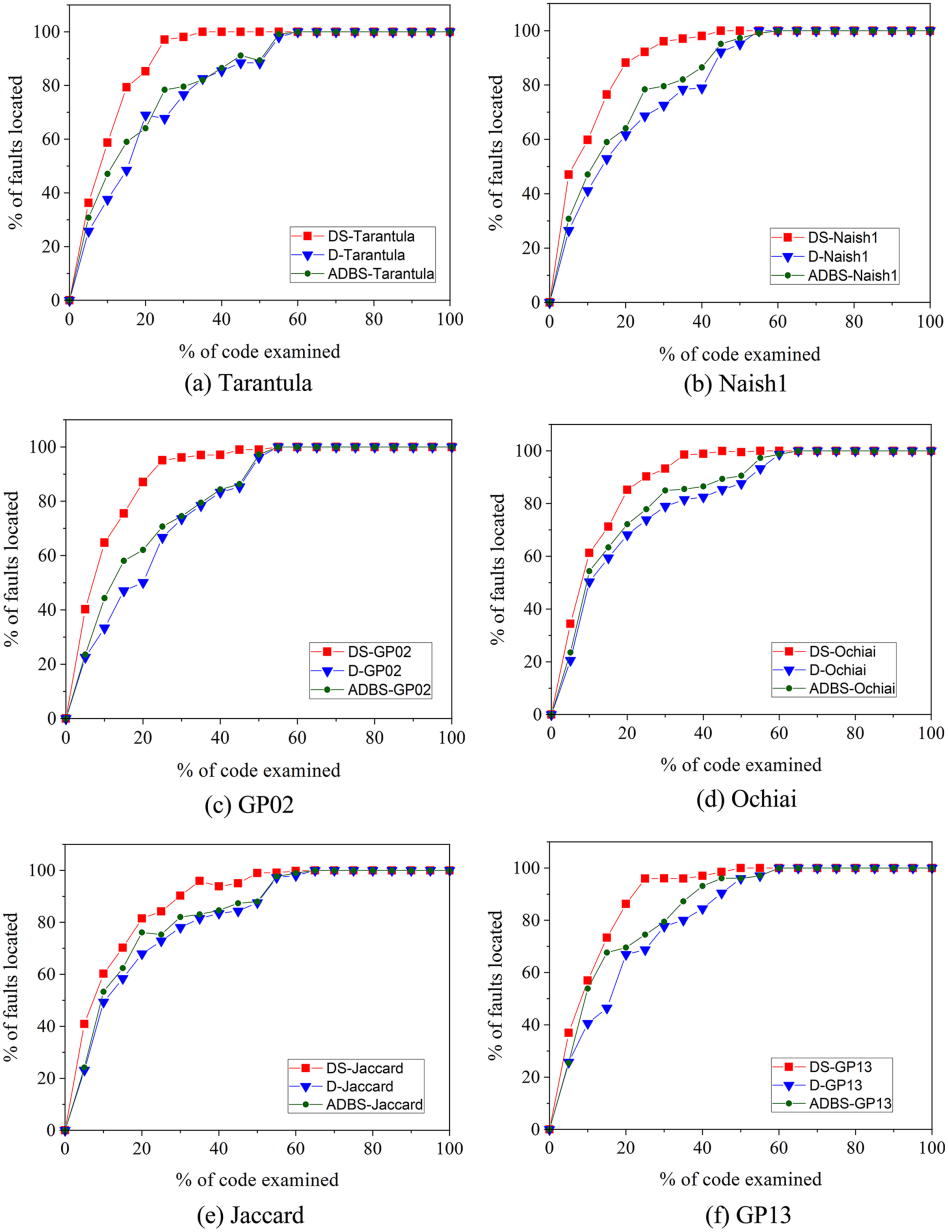
EXAM score-based comparison between original, improved and ADBS dynamic slicing.

Additionally, to more intuitively show the improvement of our approach over the compared techniques, we use the Exam metric to evaluate the effectiveness. As shown in Fig. 3, the horizontal coordinate indicates the code examined rate and the vertical coordinate indicates faults located rate. For the same code examined rate, a higher percentage of faults located rate means more effective. In Fig. 3, shows that the line of the improved dynamic slice technique is always beyond the line of the original dynamic slice and ADBS techniques, which indicates that DS-Tarantula, DS-Naish1, DS-GP02, DS-Ochiai, DS-Jaccard and DS-GP13 outperform the original and ADBS approaches in the effectiveness of fault localization. Jones & Harrold (2005); Jones, Harrold & Stasko (2002) concluded that the lower code examined rates better reflects the effectiveness of fault localization approaches. In this article, we compare the percentage of faults located by different approaches with 20% code examined rate. The faults located rate of DS-Tarantula is 85.26% compared to 68.98% of D-Tarantula and 64.06% of ADBS-Tarantula. DS-Naish1 is 88.24% compared to 61.73% of D-Naish1 and 63.70% of ADBS-Naish1. DS-GP02 is 87.10% compared to 50.12% of D-GP02 and 62.12% of ADBS-GP02. DS-Ochiai is 85.19% compared to 68.21% of D-Ochiai and 69.20% of ADBS-Ochiai. DS-Jaccard is 81.53% compared to 67.94% of D-Jaccard and 67.19% of ADBS-Jaccard. DS-GP13 is 86.26% compared to 66.97% of D-GP13 and 69.61% of ADBS-GP13. This improvement in the accuracy of fault localization is mainly due to the improved dynamic slice to narrow down the range of fault localization. It can be obviously seen that our technique has improved the accuracy of fault localization than these compared techniques.

### Experiments using Top-N metric

**Figure 4:**
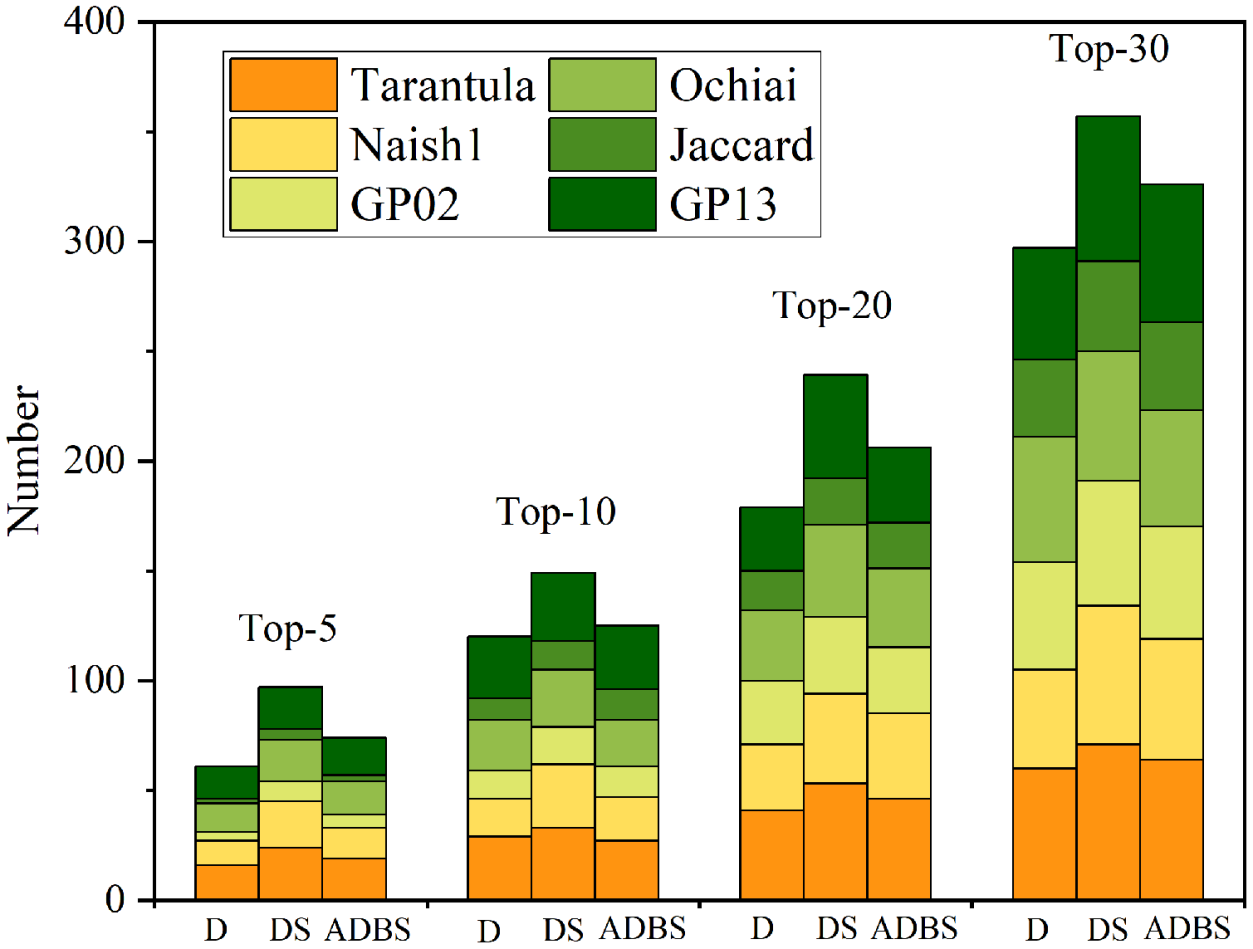
Experiments of locating all faults. Five metrics ( Top-5, Top-10, Top-20, Top-30 ) are used to test the three slicing approaches.

As shown in Table 5, Five metrics (Top-5, Top-10, Top-20, Top-30) are used to verify the accuracy and effectiveness of the D-SBFL, DS-SBFL, and ADBS-SBFL approaches. Specifically, there are 24 programs that DS-Tarantula succeeds in locating all faults by considering only the top five positions in ranked lists. When considering only the top five positions in the ranking list, DS-Naish1, DS-GP02, DS-Ochiai, DS-Jaccard13, and DS-GP13 locate 21, 9, 19, 5, and 19 programs, respectively. Moreover, the number of programs in which all faults are successfully located by DS-SBFL is always more than the others when using the other three Top- N metrics. Figure 4 shows that DS-SBFL outperforms other baseline approaches in locating all faults when using four Top-N metrics in a more intuitive way.

**Table 5:** Number of programs that all faults are successfully located.

Techniques		Top-5	Top-10	Top-20	Top-30
Tarantula	D	16	29	41	60
	DS	24	33	53	71
	ADBS	19	27	46	64
Naish1	D	11	17	30	45
	DS	21	29	41	63
	ADBS	14	20	39	55
GP02	D	4	13	29	49
	DS	9	17	35	57
	ADBS	6	14	30	51
Ochiai	D	13	23	32	57
	DS	19	26	42	59
	ADBS	15	21	36	53
Jaccard	D	2	10	18	35
	DS	5	13	21	47
	ADBS	3	14	21	40
GP13	D	15	28	29	51
	DS	19	31	47	66
	ADBS	17	29	34	63

### The comparison of time cost

In this section, we evaluate the time cost of the improved slice, the ADBS slice, and the original slice fault localization using the tarantula formula. As shown in Table 6, the time comparison between the improved slice, the ADBS slice, and the original slice fault localization method when running all fault versions on each test program. Column 2 “Collecting slice” represents the time to build slice information and collect coverage information on all test cases, the unit is second (s); Column 3 “Computing suspiciousness” represents The time of the calculation of suspiciousness; Column 4 “Total” represents the total time cost. From the data in column 2, the time of the computing slice based on the original slice is slightly longer than that of the improved slice. As can be seen from the last column of Table 6, the total time of improved slice-based fault localization is shorter than the original slice-based fault localization methods, and the difference between the improved slice and the ADBS slice is small in time overhead.

**Table 6:** The comparison of time cost.

Subject	Collecting slice			Computing suspiciousness			Total time		
	D.(s)	DS.(s)	ADBS.(S)	D.(s)	DS.(s)	ADBS.(S)	D.(s)	DS.(s)	ADBS.(S)
jtcas	5,110.94	4,800.02	4,813.56	146.56	125.03	141.57	5,257.5	4,925.05	4,955.13
tot_info	2,220.83	1,425.68	1,450.22	95.14	86.09	100.63	2,315.97	1,511.77	1,550.85
schedule	9,468.51	8,345.56	8,135.97	513.45	406.68	425.22	9,981.96	8,752.24	8,561.19
schedule2	26,944.5	24,350.5	24,360	2,655.59	2,531.24	2,550.78	29,600.08	26,881.7	26,910.77
print_tokens	26,491.7	26,355.3	26,377	1,510.36	1,236.16	1,224.62	28,002.04	27,591.5	27,601.63
print_tokens2	53,241.4	52,194.2	52,220.8	3,619.67	3,100.45	3,126.99	56,861.05	55,294.7	55,347.76
NanoXML v1	3,492.15	2,528.91	2,575.92	92.03	90.08	127.09	3,584.18	2,618.99	2,703.01
NanoXML v2	3,146.44	2,304.12	2,358.76	103.52	101.09	125.73	3,249.96	2,405.21	2,484.49
NanoXML v3	2,943.91	2,297.41	2,335.8	97.75	96.05	134.53	3,041.66	2,393.46	2,470.33
NanoXML v5	3,377.48	2,460.32	2,391.5	99.47	99.1	106.77	3,476.95	2,559.42	2,498.27
XML-sec v1	5,504.21	4,402.19	4,430.57	194.35	189.12	175.93	5,698.56	4,591.31	4,606.5
XML-sec v2	5,948.46	4,607.22	4,517.89	202.29	195.02	205.69	6,150.75	4,802.24	4,723.58
JFreeChart	2,864.79	2,102.08	1,924.31	133.92	127.58	149.81	2,998.71	2,229.66	2,074.12
Joda-time	9,132.39	8,423.33	8,417.83	472.34	458.19	474.52	9,604.73	8,881.52	8,892.35
Mockito	8,537.62	7,429.6	7,462.94	591.53	589.6	622.94	9,129.15	8,019.2	8,085.88

### Threats to validity

In this subsection, we discuss the potential threats to the validity of our empirical study.

Threats to external validity are about whether the observed experimental results can be generalized to other subjects. To guarantee the representativeness, we choose a large number of programs from the widely used Simens, Linux, and Defects4J suites. Our experiments consider both C and Java programs and include both seeded and real bugs. We realize that there is no perfect empirical study and there must be some tricky programs and bugs not considered in our experiments. We plan to enlarge our subjects in our future work.

The internal validity of this work lies in the accuracy of the slicing result computed by our approach. To avoid faults in our tool implementation, we prepared our data carefully and tested our approach with simple programs.

Threats to construct validity are about whether the performance metrics used in the empirical studies reflect the real-world situation. In the experiment, we used the cumulative number of statements examined, Cost, EXAM, and Top-N other indicators. We realize some indicators were not considered in our experiments. Therefore, using more widely metrics is our future work.

## Related work

### Slicing-based fault localization techniques

After Weiser (1982) first proposed program slicing for fault localization. Researchers have proposed various fault localization methods based on program slicing methods. The first type of method is that the program slicing results are directly used for fault localization. For example, Zhang et al. (2005) proposed a dynamic slicing-based fault localization method, which used forward computation to calculate and compare the fault localization effects of data slicing, full slicing and correlation slicing, and experimentally proved that the fault localization effects of full slicing and correlation slicing are better. Jiang et al. (2012) used the application slicing technique to locate null pointer exceptions by combining real-time stack information and performing null pointer and *alias* analysis on sliced programs. The second type of method is the combination of program slicing and program spectrum methods for fault localization. For example, Wen et al. (2011) used dynamic slicing to extract dependencies between program elements, reduce program execution traces, and then constructed a Tarantula-like formula to count statement suspiciousness on statement spectrum information. Alves et al. (2011) used dynamic slicing and modification impact analysis to improve the efficiency of Tarantula’s fault localization. In contrast to the literature (Wen et al., 2011; Alves et al., 2011), this article uses an improved dynamic slicing technique to improve the dynamic slicing. Yu et al. (2011) used the Tarantula formula to generate the priority order of suspiciousness statements based on coverage information and static control flow graphs. Yu et al. (2011) constructed the execution flow graph based on coverage information and static control flow graphs to obtain semi-dynamic slicing. Lei et al. (2012) used the intersection of static backward slicing and execution slicing to form an approximated dynamic slice, and then counted the suspiciousness of the statements. Ghosh & Singh (2021a) presented using context-sensitive slicing to reduce time and be more precise for spectrum fault localization.

### Spectrum-based fault localization approaches

Spectrum-based tuning is one of the most popular tuning techniques in the automated tuning process. The spectral information of program execution is used to calculate the suspiciousness of each statement (Yoo, 2012), Ochiai (Abreu, Zoeteweij & Van Gemund, 2007), Jaccard (Chen et al., 2002). The goal of all these methods is to assign the flawed statement the highest possible suspect value and the correct one the lowest possible suspect value. Suspiciousness ranking can help programmers save debugging time (Naish, Lee & Ramamohanarao, 2011). Renieres & Reiss (2003) proposed the nearest neighbor query error localization method. The method assumes that there is one failed run and multiple successful runs, and uses the distance metric method to count the most similar program spectrum from the successful run to the failed run, compare the differences, remove the statements executed by both failed and successful runs, and generate error locations Report. By constructing N-length execution sequences, Nessa et al. (2008) used statistical information association to mine the association between N-length execution subsequences and execution results, and then proposed an error localization method combining N-length sequences and association rules. Ribeiro et al. (2018) proposed a spectrum-based Java program fault location tool JAGUAR. It relies on data flow strategies for fault location and visualization of suspicious statement lists. Zhang et al. (2017) proposed a PageRank-based fault location, which uses the PageRank algorithm to improve the technique of spectrum-based fault location. Wong et al. (2013) proposed DStar, a technique that computes a suspiciousness score for each program statement. DStar is a well-known statistical formula used in SBFL techniques to reveal problematic statements. Xiaobo et al. (2021) proposed an entropy-based framework Efilter to filter unlabeled test cases, constructing a sentence-based entropy and a test-suite-based entropy to measure the localization uncertainty of a given test suite. To improve the effectiveness of fault localization, Wang, Wu & Liu (2021) used a clustering-based technique to identify coincidentally correct test cases from passed test suites and empirically quantified the accuracy of identifying coincidentally correct test cases to assess its effectiveness. A framework for fault localization using a multivariate logistic regression model that combines static and dynamic features collected from the program being debugged by Ju et al. (2020). Ghosh & Singh (2021b) proposed an automated framework using chaos-based genetic algorithm for multi-fault localization based on SBFL technique.

In SBFL, ranking is based solely on the suspiciousness score of each element. Elements with high scores are placed at the beginning of the list, and elements with low scores are placed at the bottom. Heiden et al. (2019) argue that the SBFL technique only exploits the program spectrum as an abstraction for program execution without considering any other useful contextual information. Vancsics et al. (2021) addresses this problem by using method call frequency. He refines the standard SBFL formula with the frequency with which the investigated method appears in call stack instances. Zou et al. (2019) considered the execution time of different techniques and thus combined different techniques. He proposes a combined technique, CombineFL, which can be configured according to time cost, and CombineFL significantly outperforms independent techniques. Furthermore, in their work, they obtain fault localization reports on program coverage information, but we propose an improved dynamic slicing that can narrow the fault scope to improve the effectiveness of fault localization.

To enhance the impact of failed test cases on fault localization, Xie et al. (2022) used a universal data augmentation method that generates synthesized failing test cases from reduced feature space for improving fault localization. Zeng et al. (2022) introduced a probabilistic approach to model program semantics and utilize information from static analysis and dynamic execution traces for fault localization, which balance could be reached between effectiveness and scalability. Wang et al. (2022) investigated the performance of Mutation-based fault localization with First-Order-Mutants and Higher-Order-Mutants on single-fault localization and multiple-fault localization.

## Conclusions and future work

In this article, we have proposed the improved dynamic slices to improve the effectiveness of fault localization. In details, we collect the dynamic slice information of program execution. Secondly, we use dynamic slices and execution results to build a hybrid spectrum. Last, a fault localization report is finally obtained by computing the suspiciousness of each statement in the hybrid slice spectrum. In the empirical study, our approach is compared with three fault localization approaches. Experimental results indicate that our approach outperforms other compared approaches. Furthermore, it can reduce approximately 1% to 17.27% of the average cost of examined code significantly. In the future, we will continue to verify the performance of our proposed technique on more subjects and further improve the accuracy of fault localization.

In the future, we want to further consider the following issues. Firstly, we plan to apply our approach to locate multiple faults. Secondly, we want to use the methods like deep learning to deal with the massive information of slice computing, so that we can improve our method to locate faults in more large-scale and real-world applications. Last but not least, we want to apply our approach to more subjects written by other programming languages such as C++, Python and conduct more detailed empirical studies.

## Supplemental Information

10.7717/peerj-cs.1071/supp-1 Supplemental Information 1 Experimental data.

10.7717/peerj-cs.1071/supp-2 Supplemental Information 2 Source code.

10.7717/peerj-cs.1071/supp-3 Supplemental Information 3 Data analysis.

## Additional Information and Declarations

### Data Availability

The following information was supplied regarding data availability: The raw data and source code are available in the Supplemental Files.
